# Metabolic dysfunction-associated steatohepatitis is the leading indication for adult liver transplantation in Saudi Arabia

**DOI:** 10.1371/journal.pone.0338438

**Published:** 2025-12-10

**Authors:** Saleh A. Alqahtani, Shadan AlMuhaidib, Dimitri A. Raptis, Waleed K. Al-Hamoudi, K. Rajender Reddy, Dieter C. Broering, Saad A. Alghamdi, The OTCE Collaborative

**Affiliations:** 1 Liver, Digestive, and Lifestyle Health Research Section, and Organ Transplant Center of Excellence, King Faisal Specialist Hospital and Research Center, Riyadh, Saudi Arabia; 2 Division of Gastroenterology and Hepatology, Weill Cornell Medicine, New York, New York, United States of America; 3 Liver, Digestive, and Lifestyle Health Research Section, and Biostatistics, Epidemiology, and Scientific Computing Department, King Faisal Specialist Hospital and Research Center, Riyadh, Saudi Arabia; 4 Organ Transplant Center of Excellence, King Faisal Specialist Hospital and Research Center, Riyadh, Saudi Arabia; 5 Liver Disease Research Center, College of Medicine, King Saud University, Riyadh, Saudi Arabia; 6 Division of Gastroenterology and Hepatology, University of Pennsylvania, Philadelphia, United States of America; Al-Azhar University, EGYPT

## Abstract

**Background:**

Liver transplantation (LT) represents the life-saving treatment for advanced liver disease. We aim to investigate LT indication trends and outcomes in Saudi Arabia, following the evolution of effective therapies for hepatitis C virus (HCV) and the rising fatty liver disease prevalence.

**Methods:**

We retrospectively analyzed data from adult patients who underwent LT from 2011 to 2023 at a tertiary referral center in Saudi Arabia. We assessed demographics, LT indication trends, Model for End-stage Liver Disease (MELD) scores, donor type, and survival outcomes.

**Results:**

A total of 1,419 patients were included. The median age was 56.9 years, with 37.4% female. Living donor LT (LDLT) represented 79.8% of all transplants, and 22.0% of recipients had hepatocellular carcinoma (HCC). Metabolic dysfunction-associated steatohepatitis (MASH) was the predominant indication for LT (33.2%), followed by HCV (18.0%) and hepatitis B virus (HBV) (17.1%). Overall survival rates at 1-, 2-, 3-, 5-, and 10-years post-transplantation were 87.9%, 85.0%, 82.4%, 77.7%, and 71.3%, respectively. Hazard ratios (HR) for mortality were lower in patients with HBV compared to MASH (HR: 0.44, 95% CI: 0.28–0.69, p < 0.001), and higher in patients aged ≥65 years (HR: 1.37, 95% CI: 1.02–1.84, p = 0.036), those with diabetes (HR: 1.33, 95% CI: 1.03–1.73, p = 0.029), and those with increased MELD score (HR: 1.02, 95% CI: 1.00–1.04, p = 0.022). LDLT was associated with reduced mortality risk (HR: 0.68, 95% CI: 0.51–0.92, p = 0.013).

**Conclusions:**

MASH represents the leading indication for LT in this large cohort, necessitating preventive strategies and early detection efforts.

## Introduction

Liver transplantation (LT) represents a life-saving treatment for patients with advanced liver disease, significantly improving survival and quality of life [1,2]. In recent decades, indications for LT have evolved, influenced by advancements in medical treatments and changes in disease prevalence [3]. Traditionally, viral hepatitis, especially hepatitis C virus (HCV) and hepatitis B virus (HBV), were the leading indications for LT [3,4]. However, the landscape has changed significantly with the advent of HBV vaccination programs and effective antiviral therapies, particularly direct-acting antivirals (DAA) for HCV, which have markedly decreased the incidence of HCV-related cirrhosis [5–7].

Parallel to the decrease in viral hepatitis-related liver diseases, there has been a notable rise in metabolic disorders, primarily driven by the global epidemic of obesity and type 2 diabetes [3,8–10]. This change has led to an increase in metabolic dysfunction-associated steatotic liver disease (MASLD) and its progressive form, metabolic dysfunction-associated steatohepatitis (MASH) [9]. MASH is now recognized as a significant contributor to liver cirrhosis and liver failure, and is increasingly overtaking viral hepatitis as the leading indication for LT [3,11,12].

In Saudi Arabia, as in many other countries, the epidemiological transition towards metabolic liver diseases has profound implications for healthcare delivery and LT outcomes. The increasing prevalence of MASH necessitates a thorough understanding of its impact on LT trends and outcomes [5,13].

This study aims to comprehensively analyze LT indications and outcomes over the past decade at a tertiary referral center in Saudi Arabia. Specifically, it focuses on the rise of MASH as the predominant indication for LT and examines its implications for patient and graft survival. By highlighting these trends, this study seeks to inform healthcare strategies aimed at prevention, early detection, and optimal management of MASH.

## Methods

### Study design and participants

We conducted a retrospective cohort study of LT recipients at a tertiary LT center from January 2011 to December 2023.

### Inclusion and exclusion criteria

#### Inclusion criteria.

Adults (≥18 years) who underwent LT at King Faisal Specialist Hospital & Research Center (KFSHRC) in Riyadh during the study period were included. Eligible patients had documented primary LT indications, including MASH, HCV, HBV, autoimmune hepatitis (AIH), biliary diseases, metabolic or genetic disorders, alcoholic liver disease (ALD), acute liver failure (ALF), transplant-related factors, and other etiologies. Data collected included demographics, comorbidities, laboratory results, and survival outcomes.

#### Exclusion criteria.

Pediatric patients (<18 years) were excluded. A flowchart of the study cohort selection is presented in S1 Fig.

### Data collection

We collected data on patient demographics (age, sex, body mass index [BMI]), primary indications for LT, hepatocellular carcinoma (HCC) status, comorbidities (heart disease, diabetes, and hypertension), laboratory values (including Model for End-stage Liver Disease [MELD] scores at the time of transplantation), perioperative details (donor type: living or deceased), and survival outcomes (patient and graft survival data at 1-, 2-, 3-, 5-, and 10-year intervals post-transplantation). When multiple etiologies were present, the primary LT indication was assigned based on the condition most likely responsible for advanced liver disease. MASH was diagnosed based on the presence of steatohepatitis, at least one cardiometabolic risk factor, and exclusion of other causes of liver disease [14].

### Outcomes

Primary outcomes focused on identifying the leading LT indications over time. Secondary outcomes assessed overall patient survival and graft survival at 1-, 2-, 3-, 5-, and 10-year intervals post-transplantation. Tertiary outcomes involved comparing outcomes between LT recipients with and without HCC. Patient survival was defined as the duration from LT to death from any cause, while graft survival was defined as the time from transplantation to graft failure, necessitating re-transplantation or resulting in death. Additionally, we analyzed survival rate differences based on primary indications for transplantation and donor type.

### Statistical analysis

We conducted the statistical analysis using IBM SPSS version 29.0 (IBM Corp., Armonk, NY, USA). Categorical variables were reported as frequencies and percentages, while continuous variables were summarized using median and interquartile range (IQR) values (25th to 75th percentiles). For the age variable, the range was also provided. Descriptive analyses were performed to provide an overview of the dataset. Figures illustrating the number and percentages of liver transplant patients by primary indication over the years were generated using Microsoft Excel version 16.88.

For comparative analysis, we utilized Mann-Whitney U tests to compare continuous variables between groups and chi-square tests for categorical variables. Kaplan-Meier survival analysis estimated patient and graft survival rates post-LT. Differences in survival curves between groups were assessed using log-rank tests. Cox proportional hazards regression analysis identified factors associated with overall survival post-LT. For the univariable analysis, variables were selected based on clinical relevance. Those found statistically significant in the univariable analysis were included in the final multivariable model. Hazard ratios (HR) with corresponding 95% confidence intervals (CI) were reported. Statistical significance was set at a threshold of p ≤ 0.05 for all tests.

### Ethics statement

The study was approved by the Institutional Review Board (IRB) at KFSHRC in Riyadh, Saudi Arabia (IRB approval number: RAC 2121012). It was conducted in accordance with the Declaration of Helsinki, local guidelines, IRB recommendations, and institutional regulations. Due to the retrospective nature of the study, the need to obtain informed consent was waived by the IRB. Data were accessed on 13/03/2024 from the institutional liver transplant registry by authorized study investigators. While the dataset included coded patient identifiers for linkage and validation purposes, all analyses were performed on de-identified data to ensure patient confidentiality.

## Results

### Patient characteristics

The characteristics of LT recipients, categorized by primary indications and ranked by the number of LTs from 2011 to 2023, are presented in Table 1. Among the 1,419 recipients, the most common indication for LT was MASH at 33.2%, followed by HCV at 18.0%, HBV at 17.1%, AIH at 9.8%, biliary diseases at 5.8%, metabolic and genetic diseases at 5.8%, and other less common indications (5.6% to 0.3%). ALD represented 2.4% of the cohort. The distribution of recipients varied over different periods, with a notable increase in transplants from 2020 to 2023, accounting for 48.5% of all transplants.

**Table 1:** Characteristics of LT recipients, overall and by primary indication from 2011–2023 (Ranked from left to right by the number of LT by primary indication).

Factors	Overall	MASH	HCV	HBV	AIH	Biliary	Metabolic & Genetics	Others	ALD	ALF	Transplant-related
**LT, n (%)**	1,419	471 (33.2)	255 (18.0)	243 (17.1)	139 (9.8)	83 (5.8)	82 (5.8)	79 (5.6)	34 (2.4)	29 (2.0%)	4 (0.3)
**Time period/Era, n (%)**											
2011-2013	184 (13.0)	51 (10.8)	63 (24.7)	42 (17.3)	12 (8.6)	3 (3.6)	6 (7.3)	7 (8.9)	0 (0.0)	0 (0.0)	0 (0.0)
2014-2016	233 (16.4)	56 (11.9)	61 (23.9)	48 (19.8)	23 (16.5)	15 (18.1)	7 (8.5)	18 (22.8)	3 (8.8)	2 (6.9)	0 (0.0)
2017-2019	314 (22.1)	105 (22.3)	43 (16.9)	58 (23.9)	28 (20.1)	14 (16.9)	38 (46.3)	13 (16.5)	1 (2.9)	11 (37.9)	3 (75.0)
2020-2023	688 (48.5)	259 (55.0)	88 (34.5)	95 (39.1)	76 (54.7)	51 (61.4)	31 (37.8)	41 (51.9)	30 (88.2)	16 (55.2)	1 (25.0)
**Age, Median (IQR)**	56.9 (46.3–63.2)	59.9 (54.8–65.1)	59.8 (54.2–64.9)	56.0 (50.4–61.9)	37.9 (30.1–50.1)	46.0 (34.2–54.5)	32.8 (24.5–58.3)	60.5 (42.0 −66.0)	56.5 (43.5–62.1)	32.3 (26.1–39.0)	42.9 (20.9–70.9)
**Age, Range**	18.0–85.0	19.3–80.0	18.0–67.3	20.0–74.9	18.2–72.0	18.8–72.6	18.0–85.0	18.0–73.5	34.0–73.7	19.7–63.0	18.0–79.8
**≥ 65 years, n (%)**	290 (20.4)	131 (27.8)	66 (25.9)	45 (18.5)	2 (1.4)	3 (3.6)	10 (12.2)	26 (32.9)	6 (17.6)	0 (0.0)	1 (25.0)
**Female, n (%)**	530 (37.4)	152 (32.3)	132 (51.8)	51 (21.0)	80 (57.6)	47 (56.6)	35 (42.7)	15 (19.0)	0 (0.0)	16 (55.2)	2 (50.0)
**BMI, Median (IQR)**	26.3 (23.0–30.7)	27.6 (24.2–32.0)	27.7 (24.4–31.2)	27.1 (23.6–31.3)	24.0 (21.3–27.4)	23.4 (20.4–26.0)	23.9 (21.0–27.0)	25.8 (22.0–29.0)	25.4 (22.0–29.1)	25.9 (22.7–31.3)	27.2 (22.5–32.5)
**BMI group, n (%)**											
Underweight	67 (4.7)	9 (1.9)	6 (2.4)	7 (2.9)	15 (10.8)	10 (12.0)	10 (12.2)	4 (5.1)	4 (11.8)	2 (6.9)	0 (0.0)
Normal	481 (33.9)	132 (28.0)	66 (25.9)	78 (32.1)	65 (46.8)	48 (57.8)	38 (46.3)	29 (36.7)	12 (35.3)	11 (37.9)	2 (50.0)
Overweight	457 (32.2)	168 (35.7)	94 (36.9)	75 (30.9)	35 (25.2)	17 (20.5)	22 (26.8)	30 (38.0)	10 (29.4)	6 (20.7)	0 (0.0)
Obesity (class 1)	279 (19.7)	103 (21.9)	59 (23.1)	59 (24.3)	15 (10.8)	6 (7.2)	7 (8.5)	16 (20.3)	6 (17.6)	6 (20.7)	2 (50.0)
Obesity (class 2)	97 (6.8)	37 (7.9)	20 (7.8)	22 (9.1)	8 (5.8)	1 (1.2)	5 (6.1)	0 (0.0)	2 (5.9)	2 (6.9)	0 (0.0)
Obesity (class 3)	38 (2.7)	22 (4.7)	10 (3.9)	2 (0.8)	1 (0.7)	1 (1.2)	0 (0.0)	0 (0.0)	0 (0.0)	2 (6.9)	0 (0.0)
**LT from living donor, n (%)**	1,132 (79.8)	387 (82.2)	182 (71.4)	197 (81.1)	116 (83.5)	67 (80.7)	68 (82.9)	70 (88.6)	23 (67.6)	19 (65.5)	3 (75.0)
**MELD score, Median (IQR)**	22.0 (17.0–24.0)	21.0 (17.0–24.0)	21.5 (16.0–22.0)	22.0 (17.0–23.0)	22.0 (17.0–27.0)	20.0 (14.0–25.0)	22.0 (17.0–26.0)	22.0 (17.5–22.0)	27.0 (18.0–32.0)	32.0 (23.5–40.0)	22.0 (20.5–22.5)
**HCC candidate, n (%)**	312 (22.0)	85 (18.0)	94 (36.9)	88 (36.2)	2 (1.4)	5 (6.0)	5 (6.1)	29 (36.7)	3 (8.8)	1 (3.4)	0 (0.0)
**Recipients’ readmission within 3 months, n (%)**	304 (21.4)	99 (21.0)	57 (22.4)	52 (21.4)	35 (25.2)	18 (21.7)	15 (18.3)	14 (17.7)	9 (26.5)	5 (17.2)	0 (0.0)
**Heart disease, n (%)**	106 (7.5)	60 (12.7)	17 (6.7)	10 (4.1)	5 (3.6)	3 (3.6)	6 (7.3)	3 (3.8)	2 (5.9)	0 (0.0)	0 (0.0)
**Diabetes, n (%)**	487 (34.3)	202 (42.9)	113 (44.3)	83 (34.2)	21 (15.1)	14 (16.9)	22 (26.8)	21 (26.6)	6 (17.6)	3 (10.3)	2 (50.0)
**Hypertension, n (%)**	720 (50.7)	226 (56.5)	130 (51.0)	114 (46.9)	68 (48.9)	39 (47.0)	36 (43.9)	44 (55.7)	13 (38.2)	9 (31.0)	1 (25.0)

**Abbreviations:** AIH, autoimmune hepatitis; ALD, alcoholic liver diseases; ALF, acute liver failure; BMI, body mass index; HBV, hepatitis B virus; HCC, hepatocellular carcinoma; HCV, hepatitis C virus; IQR, interquartile range; LT, liver transplant; MASH, metabolic dysfunction-associated steatohepatitis; MELD score, model for end-stage liver disease.

The median age of recipients was 56.9 years (IQR: 46.3–63.2), ranging from 18 to 85 years, with 20.4% aged 65 years and older. Age distribution differed among indications; recipients with metabolic and genetic diseases, ALF, and AIH were younger, and recipients with MASH and HCV were older. The proportion of recipients aged ≥65 years was highest in the MASH group (27.8%).

Gender distribution also varied, with 37.4% of recipients being female. The highest proportion of female recipients was in the AIH group (57.6%), while no females underwent LT due to ALD. The median BMI for the cohort was 26.3 kg/m² (IQR: 23.0–30.7), with notable differences among indications. The MASH and HCV groups had higher median BMIs compared to other groups.

Most recipients (79.8%) received LT from living donors, with slight variations across different indications. Patients with MASH were among the highest proportion of LDLT recipients (82.2%). The median MELD score at the time of transplantation was 22.0. Additionally, 22.0% of the recipients had HCC, with the highest proportions in the HCV and HBV groups. Details of missing data for the study variables are provided in Supplementary (S1 Table).

### Trends over time

**Fig 1:**
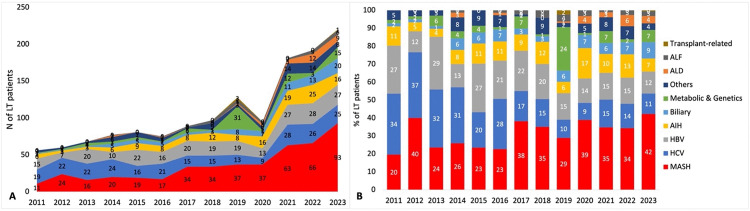
A) Number of liver transplant patients by primary indication, 2011–2023. B) Percentage of liver transplant patients by primary indication, 2011–2023. **Abbreviations:** AIH, autoimmune hepatitis; ALD, alcoholic liver diseases; ALF, acute liver failure; HBV, hepatitis B virus; HCV, hepatitis C virus; LT, liver transplant; MASH, metabolic dysfunction-associated steatohepatitis; N, Number; %, percentage.

The distribution of primary indications has varied over time, with an increase in MASH-related transplants (from 11 in 2011 to 93 in 2023), raising its proportion from 20% to 42%. However, HCV- and HBV-related cases remained relatively constant, though the proportions decreased from 34% and 27% in 2011 to 11% and 12% in 2023, respectively. ALD, which was uncommon in 2011–2019 (n = 4), showed a notable rise in the most recent era, with 30 cases reported between 2020 and 2023 (Fig 1A and 1B).

Analysis across four distinct periods (2011–2013, 2014–2016, 2017–2019, and 2020–2023) revealed changes in the distribution of primary indications, reflecting variations in disease epidemiology. Over these periods, the proportion of MASH-related LT increased from 28% (2011–2013) to 38% (2020–2023), while HCV-related LT decreased from 34% to 13%, and HBV-related LT decreased from 23% to 14% (S2 Fig).

### Comparison of characteristics between LT recipients with and without HCC

Table 2 compares the characteristics of LT recipients with HCC (n = 312 [22.0%]) and without HCC (n = 1,107 [78.0%]). HCV and HBV were significantly more prevalent among patients with HCC. Patients in the HCC group were older, with a median age of 60.7 years, and had a higher proportion of recipients aged ≥65 years (29.2%) compared to non-HCC recipients (18.0%). The gender distribution varied, with a lower proportion of females among the HCC group compared to non-HCC recipients (27.2% vs. 40.2%).

**Table 2:** Characteristic of LT recipient with and without HCC (n = 1,419).

Factors	Without HCC	With HCC	P-value
	**n = 1,107**	**n = 312**	
**LT, n (%)**			**<0.001**
MASH	386 (34.9)	85 (27.2)	
HCV	161 (14.5)	94 (30.1)	
HBV	155 (14.0)	88 (28.2)	
AIH	137 (12.4)	2 (0.6)	
Biliary	78 (7.0)	5 (1.6)	
Metabolic & Genetics	77 (7.0)	5 (1.6)	
Others	50 (4.5)	29 (9.3)	
ALD	31 (2.8)	3 (1.0)	
ALF	28 (2.5)	1 (0.3)	
Transplant-related	4 (0.4)	0 (0.0)	
**Time period/Era, n (%)**			**<0.001**
2011-2013	168 (15.2)	16 (5.1)	
2014-2016	180 (16.3)	53 (17.0)	
2017-2019	240 (21.7)	74 (23.7)	
2020-2023	519 (46.9)	169 (54.2)	
**Age, Median (IQR)**	55.8 (43.6–62.7)	60.7 (54.2–65.7)	**<0.001**
**≥ 65 years, n (%)**	199 (18.0)	91 (29.2)	**<0.001**
**Female, n (%)**	445 (40.2)	85 (27.2)	**<0.001**
**BMI, Median (IQR)**	26.0 (22.8–30.6)	27.0 (24.1–30.7)	**<0.001**
**BMI group, n (%)**			**<0.001**
Underweight	63 (5.7)	4 (1.3)	
Normal	396 (35.8)	85 (27.2)	
Overweight	338 (30.5)	119 (38.1)	
Obesity (class 1)	202 (18.2)	77 (24.7)	
Obesity (class 2)	73 (6.6)	24 (7.7)	
Obesity (class 3)	35 (3.2)	3 (1.0)	
**LT from living donor, n (%)**	915 (82.7)	217 (69.6)	**<0.001**
**MELD score, Median (IQR)**	20.0 (16.0–25.0)	22.0 (22.0–22.0)	0.397
**Recipients’ readmission within 3 months, n (%)**	230 (20.8)	74 (23.7)	0.263
**Heart disease, n (%)**	81 (7.3)	25 (8.0)	0.680
**Diabetes, n (%)**	369 (33.3)	118 (37.8)	0.140
**Hypertension, n (%)**	574 (51.9)	146 (46.8)	0.115

**Abbreviations:** AIH, autoimmune hepatitis; ALD, alcoholic liver diseases; ALF, acute liver failure; BMI, body mass index; HBV, hepatitis B virus; HCC, hepatocellular carcinoma; HCV, hepatitis C virus; IQR, interquartile range; LT, liver transplant; MASH, metabolic dysfunction-associated steatohepatitis; MELD score, model for end-stage liver disease.

Patients with HCC had a higher median BMI (27.0 kg/m²) and a greater proportion of individuals with overweight and obesity, except for obesity class 3 (3.2% in non-HCC vs. 1.0% in HCC). LDLT was less common among patients with HCC (69.6% vs. 82.7% in non-HCC).

### Patient and graft survival

#### Patient survival.

**Fig 2:**
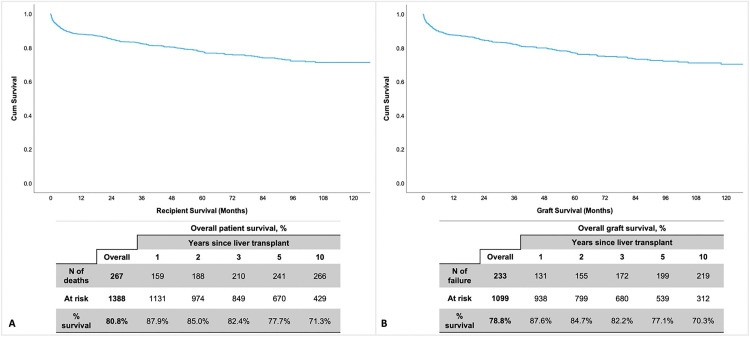
Kaplan-Meier curve for A) overall patient survival and B) overall graft survival at 1-, 2-, 3-, 5-, and 10-years post-transplantation. **Abbreviations:** N, Number; %, percentage.

The overall patient survival rates at 1-, 2-, 3-, 5-, and 10-years post-transplantation were 87.9%, 85.0%, 82.4%, 77.7%, and 71.3%, respectively (Fig 2A). The median follow-up time was 39.7 months (IQR: 13.7–81.1) for survivors and 6.0 months (IQR: 1.2–27.6) for non-survivors.

**Fig 3:**
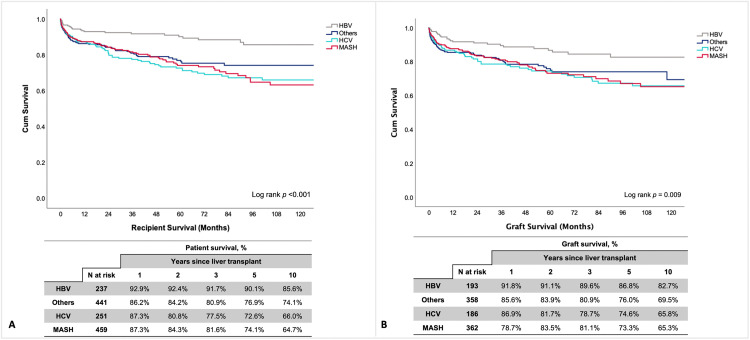
Kaplan-Meier curve for A) patient survival and B) graft survival at 1-, 2-, 3-, 5-, and 10-years post-transplantation, stratified by primary indications. **Abbreviations:** HBV, hepatitis B virus; HCV, hepatitis C virus; MASH, metabolic dysfunction-associated steatohepatitis; N, Number; %, percentage.

**Patient survival stratified by primary indications.** Survival rates at 1 and 10 years for each indication were: HBV (92.9% and 85.6%), HCV (87.3% and 66.0%), and MASH (87.3% and 64.7%). Log-rank tests showed statistically significant differences in survival between these groups (p < 0.001) (Fig 3A). Additionally**,** patient survival rates were significantly influenced by donor type (deceased vs. living donor). For deceased donor LT (DDLT), the 1- and 10-year survival rates were: HBV (90.0% and 84.4%), HCV (83.9% and 61.6%), and MASH (75.9% and 46.9%) (p < 0.001). For LDLT, the rates were: HBV (93.5% and 85.7%), HCV (88.5% and 67.9%), and MASH (89.4% and 67.2%) (p < 0.001) (S3 Fig).

#### Graft survival.

Overall graft survival rates at 1-, 2-, 3-, 5-, and 10-years post-transplantation were 87.6%, 84.7%, 82.2%, 77.1%, and 70.3%, respectively (Fig 2B). The median follow-up was 39.6 months (IQR: 16.7–79.9) for surviving grafts and 7.9 months (IQR: 1.6–37.4) for failed grafts. Stratified analysis by primary indication showed significant differences, particularly with MASH having lower rates. Graft survival rates at 1 and 10 years for each indication were: HBV (91.8% and 82.7%), HCV (86.9% and 65.8%), and MASH (78.7% and 65.3%) (p = 0.009) (Fig 3B).

**Graft survival rates stratified by donor type.** For DDLT, the survival rate differences were statistically significant despite the low number at risk (p = 0.015). For LDLT, the 1- and 10-year survival rates were: HBV (92.8% and 85.0%), HCV (87.6% and 66.9%), and MASH (88.1% and 65.4%) (p = 0.015) (S4 Fig).

### Factors associated with post-liver transplantation survival

Among 1,419 recipients, 269 (18.9%) died. Significant predictors of poorer survival included older age (≥65 years, p = 0.029), higher MELD score (p = 0.006), and diabetes (p < 0.001). In contrast, LDLT was linked to improved survival compared to DDLT (p < 0.001) (S2 Table).

The multivariable Cox regression analyses, detailed in Table 3, showed that patients with HBV had a significantly lower HR for mortality compared to those with MASH (HR: 0.44, 95% CI: 0.28–0.69, p < 0.001), and a higher HR in those aged ≥65 years (HR: 1.37, 95% CI: 1.02–1.84, p = 0.036), those with diabetes (HR: 1.33, 95% CI: 1.03–1.73, p = 0.029), and those with increased MELD scores (HR: 1.02, 95% CI: 1.00–1.04, p = 0.022). LDLT was associated with reduced mortality risk (HR: 0.68, 95% CI: 0.51–0.92, p = 0.013).

**Table 3:** Univariable and multivariable cox regression analysis of factors associated with overall post-liver transplantation survival in adults (n = 1,419).

Factors	HR (95% CI) Univariable	P-value	HR (95% CI) Multivariable	P-value
**LT primary indication**		**<0.001**		**<0.001**
MASH	1.00		1.00	
HBV	0.39 (0.25–0.61)		0.44 (0.28–0.69)	
HCV	1.07 (0.78–1.47)		1.08 (0.78–1.49)	
Others	0.92 (0.68–1.24)		1.07 (0.78–1.47)	
**Time period/Era**		0.076	–	–
2011-2013	1.00			
2014-2016	0.63 (0.44 −0.91)			
2017-2019	0.87 (0.62–1.24)			
2020-2023	0.74 (0.52–1.07)			
**Age (≥ 65 years)**	1.38 (1.05–1.82)	**0.023**	1.37 (1.02–1.84)	**0.036**
**Sex (Female)**	1.20 (0.94–1.53)	0.142	–	–
**BMI group**		0.237	–	–
Underweight	0.93 (0.52–1.65)			
Normal	1.00			
Overweight	0.79 (0.58–1.06)			
Obesity (class 1)	0.93 (0.66–1.30)			
Obesity (class 2)	0.84 (0.50–1.43)			
Obesity (class 3)	1.66 (0.91–3.03)			
**LT from living donor**	0.63 (0.48–0.82)	**<0.001**	0.68 (0.51–0.92)	**0.013**
**MELD score**	1.02 (1.01–1.04)	**0.008**	1.02 (1.00–1.04)	**0.022**
**HCC candidate**	0.73 (0.52–1.01)	0.055	–	–
**Recipients’ readmission within 3 months**	0.87 (0.64–1.17)	0.355	–	–
**Heart disease**	1.37 (0.91–2.07)	0.136	–	–
**Diabetes**	1.42 (1.12–1.81)	**0.004**	1.33 (1.03–1.73)	**0.029**
**Hypertension**	0.94 (0.74–1.20)	0.617	–	–

**Abbreviations:** BMI, body mass index; CI, confidence interval; HBV, hepatitis B virus; HCC, hepatocellular carcinoma; HCV, hepatitis C virus; HR, hazard ratio; kg/m^2^, kilograms per square meter; LT, liver transplant; MASH, metabolic dysfunction-associated steatohepatitis; MELD score, model for end-stage liver disease.

## Discussion

This study provides a comprehensive analysis of LT trends and outcomes over a 13-year period (2011–2023) at a tertiary center in Saudi Arabia, highlighting the trends in primary indications, patient demographics, and survival outcomes. Our findings reveal crucial insights into the evolving landscape of LT, with potential implications for clinical practice and future research.

Our data show that MASH is the most common indication, with approximately a 10-fold increase from 2011 to 2023, surpassing HCV and HBV. This finding reflects global trends, likely driven by the increasing prevalence of obesity, sedentary lifestyles, and metabolic syndrome, which are primary risk factors for MASH [5,15,16]. The study results are consistent with those from Western countries, where MASLD, including MASH, is the leading indication for LT due to similar lifestyle changes [15,17,18]. This necessitates targeted public health interventions and early disease management strategies to address the underlying causes.

The decrease in HCV-related transplants is attributed to the widespread availability and efficacy of DAA therapies, which have significantly reduced the burden of chronic HCV infection [6,19]. Similarly, the decline in HBV-related transplants may be due to improved vaccination coverage and antiviral treatments, reducing the progression to end-stage liver disease [20]. These trends align with previous studies showing significant reductions in HCV- and HBV-related liver disease due to medical advancements [21–23].

We also reported a unique epidemiological pattern in Saudi Arabia, which differs from Western regions where ALD predominates [21]. Religious practices and cultural norms in the Middle Eastern region strongly discourage alcohol consumption, significantly reducing ALD’s prevalence as a primary cause of end-stage liver disease [24] and uniquely shaping disease epidemiology. As previously reported, MASH is the leading cause of chronic liver diseases (CLD) in the Middle East, highlighting the low prevalence of ALD in this region [25]. Although ALD has historically been uncommon in Saudi Arabia, we observed a slight increase in the most recent era (2020–2023), from 4 to 30 cases. Despite this rise, the absolute numbers remain small, and the trend warrants further investigation to better understand potential contributing factors, including changes in clinical recognition, reporting practices, or referral patterns.

Moreover, 5.8% of our cohort underwent LT for metabolic and genetic diseases, reflecting the higher incidence of Wilson disease (WD) and progressive familial intrahepatic cholestasis (PFIC) in the region. Over the past several years, the diagnosis of WD and PFIC is mostly relied on genetic testing, as these conditions may significantly overlap with each other and with autoimmune liver diseases [26].

The age distribution aligns with the aging population and the increasing prevalence of CLD among older adults. Notably, LT recipients with MASH were older, with the highest proportion of recipients aged ≥65 years, reflecting the association between metabolic syndrome and aging [27]. Gender distribution varied significantly, with a lower proportion of female recipients overall, similar to findings reported by multiple studies worldwide. Females have lower transplant rates and higher waitlist mortality compared to males. This disparity is partly attributed to the MELD score, which systematically underestimates disease severity in females [28–30]. Females generally have smaller estimated liver volumes and weights, which contribute to size mismatch issues [29]. LT recipients with AIH had the highest proportion among females, consistent with its known predominance due to autoimmune diseases being more common in females. This finding is comparable to other studies reporting similar gender distributions in AIH [31,32]. The median BMI of the cohort was higher in the MASH group, likely reflecting the metabolic risk factors associated with this condition. This underscores the need for interventions targeting weight management and metabolic health to prevent the progression of MASH. These results are consistent with other studies that link higher BMI to MASH and its complications [9,33].

A significant proportion of the cohort consisted of patients with HCC, with higher proportions in the HCV and HBV groups, reflecting the oncogenic potential of these viral infections [34]. Patients with HCC were older, had higher BMI, and a lower proportion of females, highlighting the distinct demographic and clinical characteristics of this subgroup. These findings are consistent with global data indicating that HCC predominantly affects older males, patients with viral hepatitis, and individuals with obesity [35,36].

Significant differences were observed in survival rates among different primary indications, with LT recipients with HBV showing the highest 10-year survival rate, while those with MASH had the lowest. These differences may be influenced by underlying disease pathology, comorbidities, and response to post-transplant management. The metabolic complications associated with MASH could contribute to poorer outcomes. This is consistent with other studies indicating that metabolic liver disease is associated with higher post-transplant morbidity and mortality [15]. A previous study reported similar findings, showing that HBV survival rates are higher than those in MASH [37]. Additionally, patients with HBV-associated HCC show better survival outcomes compared to those with HCV-associated HCC in Chinese population [38].

LDLT was associated with improved survival outcomes compared to DDLT, highlighting the benefits in terms of better graft quality, reduced waiting times, and potentially fewer complications. This finding is supported by studies indicating superior outcomes with LDLT due to immediate availability and better graft quality [39,40].

Graft survival rates mirrored patient survival trends. LT recipients with MASH had the lowest graft survival rates, potentially due to the metabolic complications associated with the condition. Our analysis confirmed significant differences in graft survival based on primary indication and donor type, with LDLT showing superior outcomes, possibly due to the immediate availability and better condition of the grafts. Previous studies also report better graft survival with LDLT compared to DDLT [15,41].

Significant risk factors identified in our study include diabetes, which was associated with higher mortality rates post-transplantation. This finding aligns with global studies highlighting diabetes as a critical risk factor for poorer outcomes in LT recipients. Effective management of diabetes through integrated care models is crucial for improving post-transplant survival rates and reducing complications associated with metabolic liver diseases [42–44].

Although we report a comprehensive dataset spanning 13 years, providing robust data for trend analysis and valuable perspectives on LT epidemiology, there are limitations to consider. Specifically, being a single-center study conducted in Saudi Arabia, the generalizability of the findings may be limited. Nevertheless, it is a huge experience with relevant observations that are highly applicable to the Middle East region. Multicenter studies are warranted to validate these findings and explore variations in LT practices. Furthermore, the low prevalence of ALD and the predominance of LDLT in our center may limit extrapolation to other regions or DDLT-dominant programs. Additionally, residual confounding due to unreported perioperative or temporal factors cannot be fully excluded.

Our findings hold crucial implications for healthcare providers and policymakers, emphasizing the dominance of metabolic liver diseases in LT indications. Effective strategies integrating lifestyle modifications, early pharmacological interventions, and regular monitoring are essential to mitigate disease progression and reduce the need for transplantation. Continued research is imperative to optimize outcomes in high-risk populations and explore novel therapies for MASH and other metabolic liver diseases.

## Conclusion

MASH is the leading indication for LT in our large cohort of patients. This highlights the urgent need for healthcare agencies to implement more robust measures for the prevention and early detection of liver diseases. Effective strategies should include lifestyle modifications, early pharmacological interventions, and regular monitoring to address the metabolic risk factors associated with MASH. Additionally, continued research is essential to optimize post-transplant outcomes in high-risk populations and explore novel therapies for metabolic liver diseases. Our findings provide valuable insights that can guide clinical practice and inform healthcare policies, ultimately improving transplantation outcomes and patient survival.

## Supporting information

S1 Fig Study cohort selection flowchart. Abbreviations: DDLT, deceased donor liver transplantation; LDLT, living donor liver transplantation.
(TIF)

S1 Table Missing and available data for key factors in adult liver transplant cohort (N = 1,419). Abbreviations: DDLT: deceased donor liver transplantation; HCC: hepatocellular carcinoma; LDLT: living donor liver transplantation.
(DOCX)

S2 Fig Percentage of liver transplant patients by primary indication across different time period/Era: 2011–2013, 2014–2016, 2017–2019, 2020–2023. Abbreviations: AIH, autoimmune hepatitis; ALD, alcoholic liver diseases; ALF, acute liver failure; HBV, hepatitis B virus; HCV, hepatitis C virus; LT, liver transplant; MASH, metabolic dysfunction-associated steatohepatitis; %, percentage.
(TIF)

S3 Fig Kaplan-Meier curves for patient survival at 1-, 2-, 3-, 5-, and 10-years post-transplantation, stratified by primary indications and adjusted for a. deceased donor liver transplantation (DDLT) and b. living donor liver transplantation (LDLT). Abbreviations: DDLT, deceased donor liver transplantation; HBV, hepatitis B virus; HCV, hepatitis C virus; LDLT, living donor liver transplantation; MASH, metabolic dysfunction-associated steatohepatitis; N, Number; %, percentage.
(TIF)

S4 Fig Kaplan-Meier curves for graft survival at 1-, 2-, 3-, 5-, and 10-years post-transplantation, stratified by primary indications and adjusted for a. deceased donor liver transplantation (DDLT) and b. living donor liver transplantation (LDLT). Abbreviations: DDLT, deceased donor liver transplantation; HBV, hepatitis B virus; HCV, hepatitis C virus; LDLT, living donor liver transplantation; MASH, metabolic dysfunction-associated steatohepatitis; N, Number; %, percentage.
(TIF)

S2 Table Factors associated with overall post-liver transplantation survival in adults (n = 1,419). Abbreviations: BMI, body mass index; HBV, hepatitis B virus; HCC, hepatocellular carcinoma; HCV, hepatitis C virus; LT, liver transplant; MASH, metabolic dysfunction-associated steatohepatitis; MELD score, model for end-stage liver disease.
(DOCX)

## Abbreviations

LT: Liver transplantation
HCV: Hepatitis C Virus
MELD: Model for End-Stage Liver Disease scores
LDLT: Living donor transplantation
HCC: Hepatocellular carcinoma
MASH: Metabolic dysfunction-associated steatohepatitis
HBV: Hepatitis B Virus
HR: Hazard ratios
CI: Confidence interval
P-value: Probability value (statistical significance)
DAA: Direct-acting antivirals
IRB: Institutional Review Board
BMI: Body mass index
AIH: Autoimmune hepatitis
ALD: Alcoholic liver diseases
ALF: Acute liver failure
IQR: Interquartile range
vs.: Versus
kg/m²: Kilograms per square meter (BMI unit)
DDLT: Deceased donor liver transplantation
CLD: Chronic liver diseases
WD: Wilson disease
PFIC: Progressive familial intrahepatic cholestasis
