# Supplementary material for: Metabolic dysfunction-associated steatohepatitis is the leading indication for adult liver transplantation in Saudi Arabia
Source: PLoS One. 2025 Dec 10;20(12):e0338438. doi: 10.1371/journal.pone.0338438 (PMC12694822; doi:10.1371/journal.pone.0338438)
Supplement: S1 Table — Abbreviations: DDLT: deceased donor liver transplantation; HCC: hepatocellular carcinoma; LDLT: living donor liver transplantation. (DOCX) [file pone.0338438.s002.docx]

**S1 Table. Missing and available data for key factors in adult liver transplant cohort (N = 1,419).**

| **Factors** | **Missing, n (%)** | **Available, (%)** |
| --- | --- | --- |
| Primary indication | 0 (0.0) | 1,419 (100.0) |
| Time period/Era, n (%) | 0 (0.0) | 1,419 (100.0) |
| Age | 0 (0.0) | 1,419 (100.0) |
| Gender | 0 (0.0) | 1,419 (100.0) |
| BMI | 0 (0.0) | 1,419 (100.0) |
| Type of transplantation (LDLT vs. DDLT) | 0 (0.0) | 1,419 (100.0) |
| MELD score | 81 (5.7) | 1,338 (94.3) |
| HCC candidate | 0 (0.0) | 1,419 (100.0) |
| Recipients’ readmission within 3 months | 0 (0.0) | 1,419 (100.0) |
| Heart disease | 0 (0.0) | 1,419 (100.0) |
| Diabetes | 0 (0.0) | 1,419 (100.0) |
| Hypertension | 0 (0.0) | 1,419 (100.0) |
| Patient survival status | 0 (0.0) | 1,419 (100.0) |
| Graft survival status | 0 (0.0) | 1,419 (100.0) |

**Abbreviations:** DDLT: deceased donor liver transplantation; HCC: hepatocellular carcinoma; LDLT: living donor liver transplantation.
