# Supplementary material for: Metabolic dysfunction-associated steatohepatitis is the leading indication for adult liver transplantation in Saudi Arabia
Source: PLoS One. 2025 Dec 10;20(12):e0338438. doi: 10.1371/journal.pone.0338438 (PMC12694822; doi:10.1371/journal.pone.0338438)
Supplement: S2 Table — Abbreviations: BMI, body mass index; HBV, hepatitis B virus; HCC, hepatocellular carcinoma; HCV, hepatitis C virus; LT, liver transplant; MASH, metabolic dysfunction-associated steatohepatitis; MELD score, model for end-stage liver disease. (DOCX) [file pone.0338438.s006.docx]

**S2 Table. Factors associated with overall post-liver transplantation survival in adults (n = 1,419).**

| **Factors** | **Survived**  **n = 1,150** | **Died**  **n = 269** | **P-value** |
| --- | --- | --- | --- |
| **LT primary indication, n (%)**  MASH  HBV  HCV  Others | 377 (32.8)  219 (19.0)  189 (16.4)  365 (31.7) | 94 (34.9)  24 (8.9)  66 (24.5)  85 (31.6) | **<0.001** |
| **Time period/Era, n (%)**  2011-2013  2014-2016  2017-2019  2020-2023 | 120 (10.4)  182 (15.8)  240 (20.9)  608 (52.9) | 64 (23.8)  51 (19.0)  74 (27.5)  80 (29.7) | **<0.001** |
| **Age, Median (IQR)** | 56.7 (46.6 – 63.1) | 57.9 (48.0 – 64.4) | 0.084 |
| **Age (≥ 65 years), n (%)** | 222 (19.3) | 68 (25.3) | **0.029** |
| **Sex (Female), n (%)** | 418 (36.3) | 112 (41.6) | 0.107 |
| **BMI, Median (IQR)** | 26.3 (23.0 – 30.5) | 25.5 (22.8 – 30.7) | 0.472 |
| **BMI group, n (%)**  Underweight  Normal  Overweight  Obesity (class 1)  Obesity (class 2)  Obesity (class 3) | 53 (4.6)  380 (33.0)  383 (33.3)  227 (19.7)  81 (7.0)  26 (2.3) | 14 (5.2)  101 (37.5)  74 (27.5)  52 (19.3)  16 (5.9)  12 (4.5) | 0.152 |
| **LT from living donor, n (%)** | 940 (81.7) | 192 (71.4) | **<0.001** |
| **MELD score, Median (IQR)** | 22.0 (17.0 – 24.0) | 22.0 (17.5 – 26.0) | **0.006** |
| **HCC candidate, n (%)** | 269 (23.4) | 43 (16.0) | **0.008** |
| **Recipients’ readmission within 3 months, n (%)** | 250 (21.7) | 54 (20.1) | 0.549 |
| **Heart disease, n (%)** | 81 (7.0) | 25 (9.3) | 0.206 |
| **Diabetes, n (%)** | 359 (31.2) | 128 (47.6) | **<0.001** |
| **Hypertension, n (%)** | 583 (50.7) | 137 (50.9) | 0.945 |

**Abbreviations:** BMI, body mass index; HBV, hepatitis B virus; HCC, hepatocellular carcinoma; HCV, hepatitis C virus; LT, liver transplant; MASH, metabolic dysfunction-associated steatohepatitis; MELD score, model for end-stage liver disease.
